# Influencing Factors of Math Anxiety Among Elementary School Students

**DOI:** 10.3390/bs16030359

**Published:** 2026-03-04

**Authors:** Álvaro Antón-Sancho, Erika Cañibano-Arias

**Affiliations:** 1 Department of Mathematics and Experimental Science, Fray Luis de Leon University College of Education, C/Tirso de Molina, 44, 47010 Valladolid, Spain; 2 Faculty of Humanities and Education, Catholic University of Ávila—Universidad Católica de Ávila (UCAV), C/Canteros, s/n, 05005 Ávila, Spain; 3 PhD Program in Psychological Well-Being and Quality of Life, Pontifical University of Salamanca, C/Compañía, 5, 37002 Salamanca, Spain; ecanibanoar@upsa.es

**Keywords:** math anxiety, mathematics, general anxiety, elementary school, mathematics education

## Abstract

Math anxiety, or a student’s lack of confidence in learning mathematics, is one of the emotional dimensions with the greatest impact on mathematics education. Sociological factors such as sex and age, demographic aspects like cultural characteristics, and emotional variables such as general anxiety have been identified as significantly influencing math anxiety. This study conducts quantitative, descriptive, correlational, and regression analyses of the influence of sex, age, and general anxiety on math anxiety in a sample of 185 Spanish elementary students. It also examines whether the effects of age and general anxiety on math anxiety differ by sex. For this purpose, students’ responses to a quantitative questionnaire are analyzed. The instrument combines two validated scales: (i) STAIC T-Anxiety, measuring general anxiety, and (ii) AMAS, measuring math anxiety. Results show that students exhibit moderate average math anxiety, which is not significantly affected by sex. However, significant correlations between math anxiety, age, and general anxiety were found, independent of sex. The study highlights the need to design corrective measures for math anxiety and suggests lines for future research.

## 1. Introduction

Math anxiety is a negative emotional reaction characterized by feelings of tension, apprehension, and worry experienced by individuals when engaging with mathematical content or situations, often associated with a lack of confidence in their mathematical abilities (Ashcraft, 2002). This lack of confidence leads to negative emotional responses, in the form of nervousness, a sense of overwhelm, or blocking in the face of a problematic situation with mathematical content that is presented to them (Ashcraft, 2002). Math anxiety can affect both adults and children and is not necessarily linked to an academic context of learning mathematics (Ashcraft et al., 1998; Hembree, 1990). However, the study of math anxiety among elementary school students is of particular interest because of the impact it can have on children’s mathematics learning (Finlayson, 2014) and on the choice of higher education studies (Megreya & Al-Emadi, 2023). Despite this, studies on math anxiety in university and high school students abound but are surprisingly scarce among elementary school students (Jackson & Leffingwell, 1999).

Many students perceive mathematics as a difficult subject and report feeling anxious or worried about the subject. According to data collected by the Programme for International Student Assessment of the Organisation for Economic Co-operation and Development (OECD, 2013), about 20% of students indicate that mathematics causes them high levels of anxiety, 33% report feeling tense when faced with mathematical tasks, and 59% acknowledge being worried about the difficulty of mathematics lessons. The OECD suggests that the development of math anxiety among schoolchildren has a negative influence on their learning of mathematics. The specialized literature confirms that math anxiety has a negative impact on the academic performance of children and adolescents in mathematics (Barroso et al., 2021). Indeed, math anxiety has been shown to correlate negatively with academic performance of children and adolescents in mathematics and, in fact, with overall academic performance (Karimi & Venkatesan, 2009). However, there is some divergence in this regard in the literature. In fact, some works find that academic achievement is a distal predictor of math anxiety (St-Omer & Chen, 2023). Other research shows that the impact of math anxiety on the development of mathematical skills depends on students’ motivation towards mathematics. Thus, math anxiety would positively influence the academic performance of children in mathematics who are more motivated (Wang et al., 2015).

In any case, studies that measure math anxiety and find the factors that influence its occurrence constitute an important line of research in the field of mathematics education (Dowker et al., 2016). The reason is that these studies help to delve deeper into the mechanisms of mathematics learning and allow guiding educators in developing strategies for the development of mathematical skills (Wang et al., 2015; Dondio et al., 2023) and other transversal skills affected by math anxiety, such as memory (Ashcraft & Krause, 2007; Vos et al., 2023). Understanding the mechanisms through which math anxiety affects learning is crucial for designing effective interventions. Research has identified several cognitive pathways by which math anxiety impairs mathematical performance. First, math anxiety interferes with working memory processes, particularly the central executive component responsible for manipulating numerical information (Ashcraft & Kirk, 2001). When students experience anxiety during mathematical tasks, intrusive thoughts and worries consume cognitive resources that would otherwise be available for problem-solving (Eysenck et al., 2007). Second, math anxiety disrupts attentional control, causing students to avoid mathematical content or disengage when faced with challenging problems (Suárez-Pellicioni et al., 2016). Third, motivational factors play a mediating role. Indeed, students with high math anxiety tend to develop negative attitudes toward mathematics, reduced self-efficacy, and avoidance behaviors that further limit their exposure to mathematical learning opportunities (Wigfield & Meece, 1988). These mechanisms are not mutually exclusive and often interact in complex ways that compound the negative effects of math anxiety on learning outcomes.

Specifically, some studies reveal that incorporating less procedural and more meaningful teaching strategies helps to lower math anxiety levels and increase learning (Gutiérrez-Rubio et al., 2020). Others claim that online education would serve to reduce math anxiety levels, although this would not necessarily lead to an increase in mathematics learning (Pirrone et al., 2022). The identification of factors influencing math anxiety, such as age, grade, or the specific area of mathematics being learned, would make it possible to predict the appearance or increase of math anxiety with the aim of adjusting the didactic methodology (McMullan et al., 2012). The identification of these factors influencing math anxiety is also useful for designing instruments adapted to different age groups, educational levels, or areas of knowledge, in the case of university studies, to measure math anxiety (McKenna et al., 2022).

The literature identifies numerous factors that influence the development of math anxiety among schoolchildren. Among them, some refer to psychological aspects, such as lack of self-confidence (Finlayson, 2014) or the presence of attention deficit (Colomé et al., 2023); sociological aspects, such as sex or age (Luttenberger et al., 2018); or environmental characteristics, such as the attitude of parents (Cosso et al., 2023; Guzmán et al., 2023) or teachers (Luttenberger et al., 2018; Zanabazar et al., 2023) towards mathematics. The existence of a correlation between math anxiety and general anxiety, understood as the feeling of nervousness and worry that occurs, occasionally or constantly, without there necessarily being an objective reason for that worry, has also been proved (Hembree, 1990). This correlation, although significant, is moderate, suggesting that the phenomenon of math anxiety cannot be reduced to that of general anxiety (Hembree, 1990).

Likewise, Brown et al. (2020) conducted a comparative study on math anxiety in the USA and Colombia among university students. These authors concluded that cultural differences between the two populations lead to different levels of math anxiety. It can be concluded, therefore, that culture is a conditioning factor of math anxiety. Despite this, most of the studies on math anxiety are contextualized in the English-speaking world, and are scarce in the European Mediterranean and Latin geographical area (Ashcraft & Ridley, 2005). In fact, there is an outstanding abundance of studies on math anxiety contextualized in the USA, while the studies contextualized in other countries such as Italy, Holland, Spain, or Israel are still very much in the minority (Ersozlu & Karakus, 2019).

On the other hand, one of the most pressing needs in math anxiety research is the study of how the different influential factors mentioned above relate to each other. For example, answering questions such as whether students’ age influences math anxiety differently depending on their sex provides a future line of research raised by the literature (Dowker et al., 2016).

Despite the extensive research on math anxiety described above, several gaps remain. First, most studies have been conducted in English-speaking contexts, with limited representation of Mediterranean and Latin populations where cultural factors may shape math anxiety differently (Brown et al., 2020). Second, while age and sex have been identified as influential factors, few studies have examined how these variables interact—for instance, whether the relationship between age and math anxiety differs for males and females. Third, the connection between general anxiety and math anxiety in elementary students, and whether this relationship is moderated by sex, requires further investigation. Addressing these gaps would contribute to a more nuanced understanding of math anxiety development and inform culturally sensitive intervention strategies. The present study addresses these gaps by examining the interplay of sex, age, and general anxiety in shaping math anxiety among Spanish elementary school students.

Specifically, the general research objective of the present work is to carry out a quantitative, descriptive, correlational, and regression study that analyzes the influence of sex, age, and general anxiety on the math anxiety of a group of Spanish elementary school students. Specifically, the aim is to achieve the following specific objectives: (i) to check whether the sex, age, or general anxiety of the participants influences their levels of math anxiety; and (ii) to check whether the way in which general anxiety and age influence math anxiety is different according to the sex of the participants. Therefore, the following research questions will be answered: (i) do sex, age, and general anxiety of Spanish elementary school students influence their levels of math anxiety? and (ii) does the way in which age and general anxiety influence their math anxiety depend on the sex of elementary school students?

## 2. Literature Review

The earliest works on math anxiety relate it to general anxiety, either understood as a characteristic of one’s own personality (trait anxiety) or as a temporary episode (state anxiety; Hembree, 1990). However, it has been proven that the correlation between math anxiety and general anxiety is around 0.40, while the correlation between math anxiety measures reported by different measurement instruments is around 0.65 (Hembree, 1990). Some studies have shown that people with math anxiety express it through physiological reactions typical of general anxiety, such as sweating or accelerated heart rate (Ashcraft & Faust, 1994). However, these same studies support that this type of physiological reactions occur with some mathematical tasks, such as arithmetic computation in complex situations, but not in others, such as verbal expression situations (Ashcraft & Faust, 1994; Faust et al., 1996).

The relationship between general anxiety and math anxiety has been extensively studied, with research suggesting that while these constructs are related, they are not identical. General anxiety influences math anxiety through both direct and indirect pathways. Students with elevated trait anxiety—a stable personality characteristic reflecting a general tendency to experience anxiety—show heightened emotional reactivity to mathematical situations (Wang et al., 2014). This predisposition interacts with environmental factors, such as negative experiences with mathematics or exposure to anxious role models, to shape the development of math-specific anxiety. Hill et al. (2016) found that in elementary and secondary school students, the relationship between general anxiety and math anxiety changes with age: while both are moderately correlated in younger children, math anxiety becomes increasingly independent from general anxiety as students mature. This developmental pattern suggests that math anxiety, although initially connected to general anxious tendencies, gradually becomes a more domain-specific phenomenon influenced by accumulated experiences with mathematics. Understanding this relationship is important because interventions targeting general anxiety regulation skills may have secondary benefits for reducing math anxiety, particularly in younger students (Punaro & Reeve, 2012).

In any case, the correlation between math anxiety and general anxiety depends on whether trait anxiety or state anxiety is measured, as each usually leads to different measures. Specifically, the literature reports a higher correlation between math anxiety and trait anxiety (Goetz et al., 2013). In any case, the analysis of the influence of general anxiety on math anxiety is interesting for the purpose of measuring whether the control of general anxiety can influence math anxiety and, in turn, mathematics learning (Hill et al., 2016).

There is a wide range of math anxiety measurement instruments aimed at adults, university students, and students older than 8 years, while those instruments focused on the early development of math anxiety are scarce (Vukovic et al., 2013). Among the most widely used is the Mathematics Anxiety Rating Scale (MARS; Richardson & Suinn, 1972). The MARS is a self-report questionnaire that can be administered individually or in groups to identify levels of math anxiety and design specific interventions to help reduce it. It consists of 98 items and bases its situational anxiety ratings on a Likert-type scale from 1 to 5, where 1 represents a very low level of anxiety and 5 a very high level.

Among the adapted versions of the MARS is the Math Anxiety Rating Scale-Revised (MARS-R; Plake & Parker, 1982). This scale identifies two factors: anxiety about learning mathematics and anxiety about the mathematics learning assessment process. The Mathematics Anxiety Rating Scale for Adolescents (MARS-A; Suinn & Edwards, 1982) is an adaptation aimed at adolescents, and the Math Anxiety Rating Scale Elementary Form (MARS-E; Suinn et al., 1988) is aimed at elementary school students. From these adaptations, the Abbreviated Version of the Math Anxiety Rating Scale (AMARS; Alexander & Martray, 1989) was developed. This reduced version of the MARS consists of 25 items that analyze three factors: anxiety about mathematics tests, anxiety about numerical computations, and anxiety about the mathematics lesson. The Abbreviated Math Anxiety Scale (AMAS; Hopko et al., 2003) is the shortest and most current version of the MARS. This scale is composed of 9 items that measure math anxiety in relation to performance and evaluation, and is rated on a 5-point scale, from very low anxiety (value 1) to high anxiety (value 5). The validation of this instrument has been carried out in a large population of 1239 children and young students from very diverse geographical locations and ethnicities, by means of factor analysis and measurement of reliability (Hopko et al., 2003). It has been used in populations of a wide variety of ages and, in fact, has been specifically validated in populations of elementary school children (Caviola et al., 2017). However, the literature recognizes that this instrument presents interpretation difficulties when administered to children under 8 years of age, for whom adapted instruments are currently being developed (Primi et al., 2020).

For the study of general anxiety, different validated measurement tools have been developed, with one of the most widely used instruments in the literature being the State-Trait Anxiety Inventory (STAI; Spielberger et al., 1983). It is composed of two subscales of 20 items each: the STAI-E, which measures state anxiety, and the STAI-R, which measures trait anxiety. This instrument has an adaptation for elementary school children, the State-Trait Anxiety Inventory for Children (STAIC; Nilsson et al., 2012). This scale is intended for children of all ages, as it can be administered to them in a comprehensible manner (Nilsson et al., 2012). Again, this instrument has two subscales: STAIC S-Anxiety and STAIC T-Anxiety, which measure state anxiety and trait anxiety, respectively. These two subscales are separate and are composed of a total of 20 items measured on a 1 to 3 Likert scale.

Among the influential factors in the development of math anxiety, the most studied in the literature is sex (Hill et al., 2016). In this sense, females present higher levels of math anxiety than males in adulthood (Ferguson et al., 2015; Rossi et al., 2022). Among the studies contextualized in pre-university and high school students, there is some discrepancy regarding the influence of sex. Many of the papers find that the incidence of math anxiety is higher among females than males, as is the case among adults (Primi et al., 2014; Kvedere, 2014; Justicia-Galiano et al., 2023). The authors often attribute these differences to the existence of cultural sex stereotypes, rather than to other types of affective or personality factors (Rossi et al., 2022; Justicia-Galiano et al., 2023; Vos et al., 2023). Going deeper into these stereotypes, it can be found that females suffer greater pressure and lack of confidence than males regarding their performance in learning mathematics. This leads them to suffer greater anxiety because they feel forced to achieve good marks (John et al., 2023). In contrast, there are papers that find no differences between sexes in the math anxiety levels of adolescent students (Kyttälä & Björn, 2014).

Among the studies contextualized in elementary school students (under 12 years of age), the proportion of those that do not find differences by sex in the levels of math anxiety increases significantly. All these studies are developed in English-speaking contexts (Punaro & Reeve, 2012; Harari et al., 2013; Mitchell & George, 2022). Thus, the differences that exist between students under 12 years of age and older students suggest that age significantly influences math anxiety and the differences in this regard between females and males. However, as far as it has been possible to explore, there is no work that analyzes this influence.

On the other hand, there are studies that find differences by sex in math anxiety in elementary school students. Arnal-Palacián et al. (2022) study math anxiety in a group of 496 Spanish elementary school students, although they do not use a validated instrument adapted from the MARS, but a questionnaire designed by the authors. Moreover, their study is temporally situated in a time of confinement due to the COVID-19 pandemic, which could influence the results. They find that females present higher levels of math anxiety than males and that math anxiety is increasing in intensity with increasing grade (and, thus, age). They do not study, however, whether or not differences by sex are age-dependent.

Yu et al. (2024) analyze the impact of math anxiety on the academic performance of a very large sample of Asian elementary and secondary school students. The authors conclude that this impact is more negative among females than males and that age does not significantly influence this difference.

Few studies have been found that analyze differences by sex, both in general anxiety and math anxiety, in elementary school students. The works by Devine et al. (2012) and Hill et al. (2016) stand out. Devine et al. (2012) contextualize their research in British pupils aged 7 to 10 years. The authors find a positive correlation between math anxiety and general anxiety, with no significant differences between females and males in this correlation. They also find a negative correlation between general anxiety and academic performance in mathematics, which is higher among females than males. However, the authors do not analyze the influence of age on these correlations. For their part, Hill et al. (2016) concluded that math anxiety is higher in females than in males, regardless of educational stage. They also found a moderate positive correlation between general anxiety and math anxiety. However, they did not study differences by sex in the above correlations or the impact that the age of the participants might have on these correlations.

## 3. Materials and Methods

### 3.1. Participants and Data Collection

A total of 185 elementary school students participated in this study. These participants were chosen by a non-probabilistic convenience sampling process. Specifically, it was a group of students belonging to the same elementary school in Valladolid (Spain). The participants were specifically the students at the school who are part of the last four grades of elementary education (3rd to 6th grade, which corresponds to ages 8 to 11–12 years old). Children in the 1st and 2nd grades were excluded from the study because they were considered too young to accurately make the assessments requested in the questionnaire used as a research instrument. Both the participating students and the school principal were aware of the research objectives of the study prior to its implementation, and the participation of the students was expressly consented to in writing and was voluntary, free, and anonymous: at no time were data that could lead to the identification of participants collected.

The final sample consisted of 89 males (48.1%) and 96 females (51.9%), with a mean age of 9.61 years (SD = 1.24, median = 10 years). Participants were distributed across grades as follows: 46 in 3rd grade, 45 in 4th grade, 48 in 5th grade, and 46 in 6th grade. The age distribution was: 2 participants aged 7, 44 aged 8, 40 aged 9, 45 aged 10, 48 aged 11, 5 aged 12, and 1 aged 13. Thus, 95.7% of participants were between 8 and 11 years old, the typical age range for 3rd–6th grade elementary students in Spain. These participants outside the typical age range were retained in all analyses for the following reasons: (i) the two 7-year-olds were enrolled in 3rd grade and therefore exposed to the same curriculum as their 8-year-old peers because they are about to turn 8 years old and, based on their date of birth, they are eligible for this grade, (ii) in the same way, the 13-year-old student is receiving the same curricular-level education as the rest of the 6th-grade classmates, and (iii) excluding them would not substantively change the results given their small number relative to the total sample (*n* = 185).

Participants responded to the questionnaire in December 2022 within the school environment and school hours. Specifically, the participants, by groups corresponding to their grades, accessed the questionnaire through Google Forms^®^ (Google LLC, accessed December 2022) individually but all at the same time within each group, in the computer room of the school, and under the supervision of the authors and the teachers at the school. The supervised administration allowed researchers to clarify doubts about question interpretation, which is a methodological strength that enhances response validity. However, we acknowledge that the presence of teachers and researchers may have introduced some degree of social desirability bias, potentially leading some students to underreport anxiety levels. To minimize this influence, students were assured of complete anonymity, and supervisors maintained a neutral, non-evaluative demeanor throughout the administration. When clarification was needed, particularly regarding the term anxious, a standardized procedure was followed: supervisors explained that anxious means worried or nervous, without providing examples that might bias responses toward particular answer choices. This standardized clarification approach was documented and applied consistently across all administration sessions.

### 3.2. Variables and Hypotheses

The main explanatory variable is the sex of the participants (dichotomous nominal, with male and female values). The secondary explanatory variables are the age of the participants and their levels of general anxiety (both quantitative). General anxiety is measured on a scale from 1 to 3, where 1 indicates low anxiety and 3 indicates high anxiety. The grade of the participants was not taken as a variable because there is a correspondence between ages and grades (in the 3rd grade of elementary education, the participants are, in general, 8 years old, and in the 6th grade of elementary education, the participants are, in general, 12 years old).

The explained variable is math anxiety, which is quantitative and is measured on a scale of 1 to 5, where 1 means very low anxiety, 2 low anxiety, 3 intermediate anxiety, 4 high anxiety, and 5 very high anxiety.

Based on the reviewed literature, the present research seeks to contrast the following null hypotheses, which are grounded in previous empirical findings and theoretical frameworks:

**Hypothesis** **1** **(H1).**  *The sex of the participants does not significantly influence their mean levels of math anxiety.*

Rationale: While studies in adult and adolescent populations consistently find higher math anxiety in females (Ferguson et al., 2015; Rossi et al., 2022), research in elementary school populations shows mixed results, with several studies reporting no sex differences (Punaro & Reeve, 2012; Harari et al., 2013). This suggests that cultural stereotypes may not yet have influenced children under 12 years of age, particularly in non-English-speaking contexts.

**Hypothesis** **2** **(H2).**  *The age of the participants does not significantly influence their mean levels of math anxiety.*

Rationale: Developmental research suggests that math anxiety may change across elementary school years due to increasing curricular complexity and evolving cognitive capacities (Ashcraft & Kirk, 2001). However, the specific trajectory of this relationship in Spanish students remains unexplored.

**Hypothesis** **3** **(H3).**  *The correlation between age and math anxiety is not influenced by the sex of the participants.*

Rationale: If sex differences in math anxiety emerge gradually through cultural socialization, we might expect age to interact with sex. However, given the absence of main sex effects found in similar populations, we hypothesize no differential age effects by sex.

**Hypothesis** **4** **(H4).**  *The level of general anxiety does not significantly influence the mean levels of math anxiety of the participants.*

Rationale: Previous research has established a moderate positive correlation between general anxiety and math anxiety (Hembree, 1990; Hill et al., 2016), suggesting shared underlying mechanisms such as worry and cognitive interference. We test whether this relationship holds in a Spanish elementary school sample.

**Hypothesis** **5** **(H5).**  *The correlation between general anxiety and math anxiety is not influenced by the sex of the participants.*

Rationale: Devine et al. (2012) found no sex differences in the anxiety-math anxiety correlation among British children aged 7–10. We test whether this pattern generalizes to Spanish students.

### 3.3. Instrument

As a research instrument, two validated questionnaires were used (Appendix A): (i) State-Trait Anxiety Inventory for Children (STAIC) (Spielberger, 1973) to measure general anxiety (state); and (ii) Abbreviated Math Anxiety Scale (AMAS) (Hopko et al., 2003) to measure math anxiety. Both questionnaires are validated for use with child populations. The final instrument consists of the conjunction of both questionnaires, together with the questions on sex and age, to delimit the profile of the participants.

The STAIC questionnaire consists of two self-report subscales: the STAIC S-Anxiety, which measures transient anxiety states (state anxiety), and the STAIC T-Anxiety subscale, which measures participants’ stable personality traits for experiencing anxiety states (trait anxiety). In the present study, only the T-Anxiety subscale was used, which consists of 20 Likert-type questions from 1 to 3 asking how often the participant is in the state described in each question (1 means almost never, 2 means sometimes, and 3 means frequently). The decision to measure trait anxiety rather than state anxiety was based on theoretical and methodological considerations. Trait anxiety represents a stable personality characteristic reflecting an individual’s general tendency to experience anxiety across situations, whereas state anxiety captures temporary anxiety experienced at a specific moment (Spielberger et al., 1983). Research has shown that math anxiety correlates more strongly with trait anxiety than with state anxiety (Goetz et al., 2013), suggesting that dispositional tendencies to experience worry are more relevant to understanding math anxiety than momentary emotional states. Furthermore, trait anxiety is more closely related to the etiology and development of math anxiety over time (Wang et al., 2014), making it more appropriate for investigating the long-term factors that influence math anxiety levels. By focusing on trait anxiety, this study examines relatively stable individual differences that may predispose students to experience math anxiety, rather than transient anxiety that might vary depending on the immediate testing context.

We acknowledge that state anxiety (S-Anxiety) is also relevant to understanding anxiety in mathematical contexts, particularly during test-taking or when students face specific mathematical challenges. However, we excluded the S-Anxiety subscale for three reasons. First, our research objectives focus on identifying stable individual and demographic factors (sex, age, general anxious disposition) that predispose students to math anxiety, rather than measuring anxiety levels in a specific testing situation. State anxiety would capture students’ immediate emotional response during our questionnaire administration, which could be influenced by numerous transient factors (e.g., time of day, recent classroom events, anticipation of recess) unrelated to their typical math anxiety levels. Second, administering both STAIC subscales would have substantially increased questionnaire length, potentially causing fatigue and reduced response quality in our elementary school participants. Third, prior research has demonstrated that trait anxiety shows stronger and more stable associations with math anxiety than state anxiety (Goetz et al., 2013), making it the more theoretically relevant construct for understanding the development of math anxiety. Nevertheless, we recognize that the exclusion of state anxiety limits our ability to capture situational variability in anxiety responses, and future research might benefit from examining both trait and state anxiety to provide a more comprehensive picture of how general anxiety relates to math anxiety across different mathematical contexts and situations.

Furthermore, the use of trait anxiety as the measure of general anxiety is directly aligned with the objectives and hypotheses of the present study. As stated in the general research objective, this work seeks to analyze the influence of general anxiety on math anxiety levels—a relationship that is best captured through a stable, dispositional measure rather than a transient one. Accordingly, the STAIC T-Anxiety subscale serves as the operationalization of the secondary explanatory variable described above, where general anxiety is conceived as an enduring individual characteristic. This choice also underpins the formulation of hypotheses H4 and H5, which examine whether trait anxiety predicts math anxiety and whether this relationship differs by sex. A state anxiety measure would primarily reflect momentary emotional arousal during the assessment session itself, which could confound these relationships and would be conceptually inconsistent with the stable individual-difference framework that guides the study. The alignment between this methodological decision and the theoretical framework outlined before—which emphasizes the dispositional nature of trait anxiety and its differential role relative to state anxiety in predicting math anxiety—therefore ensures internal coherence across all components of the research design.

### 3.4. Statistical Analysis

For the analysis of the distribution of the participants by sex and grade, the goodness of fit test to a homogeneous distribution was used. For the validation of the instrument, the statistics of the confirmatory factor analysis were computed, and the internal reliability was studied using Cronbach’s alpha parameters. For the analysis of the responses, the main descriptive statistics were obtained (mean, standard deviation, and coefficient of variation) and it was verified, using the Shapiro-Wilk test, that the responses were normally distributed. For the contrast of the hypotheses, the *t*-test with Welch’s correction was used without assuming equality of variances to check if there were significant differences by sex in the levels of math anxiety. In addition, Pearson’s correlation test was performed, and polynomial regression models were analyzed to test the relationship between age and general anxiety with math anxiety. Finally, Fisher’s correlation comparison test was used to identify whether the influence of age and general anxiety on math anxiety depended on the sex of the participants. A significance level of α = 0.05 was used for all statistical tests.

## 4. Results

Statistical tests confirmed that the sample distribution was appropriate for the planned analyses. The distribution by sex was homogeneous (χ^2^ = 0.2649, *p* = 0.6068), as was the distribution by grade (χ^2^ = 0.1027, *p* = 0.8774). Additionally, there were no significant differences in sex distribution across grades (χ^2^ = 0.6823, *p* = 0.8774), indicating a balanced representation of males and females within each grade level (Table 1).

### 4.1. Instrument Validity

The confirmatory factor analysis performed on the questionnaire responses reveals the construct validity of two scales (general anxiety: items 1 to 20; math anxiety: items 21 to 29) and that the data have measurement invariance (model chi-square 236.51, *p*-value < 0.0001). The incremental fit indices are good (adjusted goodness-of-fit index 0.8690; NFI 0.8042; NNFI 0.8781; CFI 0.8909; IFI 0.8944). The absolute fit indices are also adequate (GFI 0.8939; SRMR 0.0591). Cronbach’s alpha ensures internal consistency (0.8353 for general anxiety and 0.8020 for math anxiety).

### 4.2. Responses

The participants express having intermediate-high levels (above 1.5 out of 3) of general anxiety, but intermediate-low levels (below 2.5 out of 5) of math anxiety (Table 2). However, the heterogeneity of the responses is greater in the family of questions on math anxiety, since they present greater deviations (Table 2). The Shapiro-Wilk test reveals that both families of responses (general anxiety and math anxiety) are normally distributed. The bilateral t-test for comparison of mean responses between the independent populations of males and females confirms that there are no significant differences between sexes in terms of mean general anxiety (*t*-statistic −0.2954, *p*-value 0.7677) nor in terms of mean math anxiety (*t*-statistic −0.0570, *p*-value 0.9546). This allows support for the null hypothesis H1 (sex does not significantly influence math anxiety).

**Figure 1:**
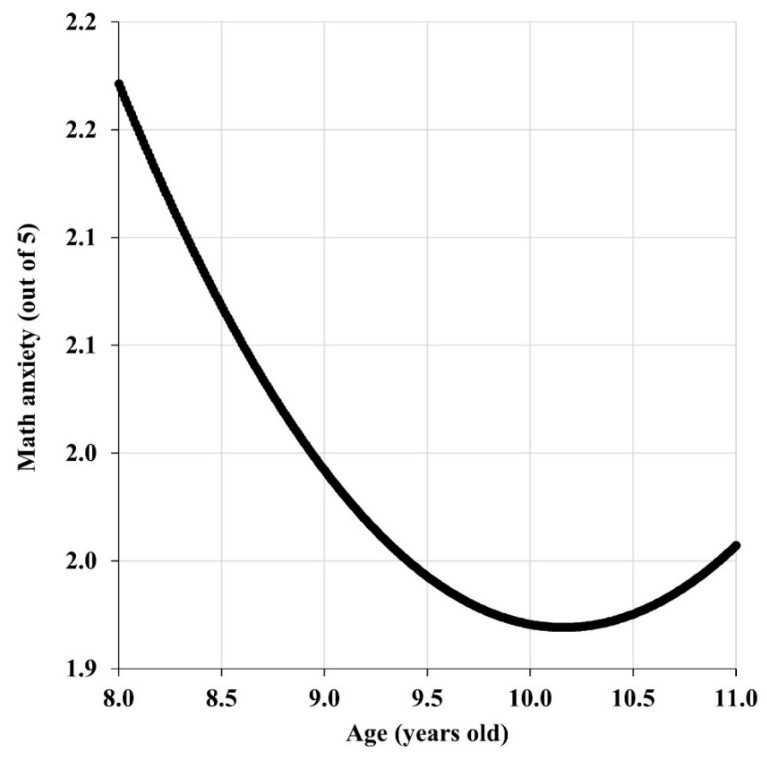
Evolution of math anxiety with age of participants.

There is a statistically significant correlation between the age of the participants and their levels of math anxiety (correlation coefficient −0.0556, *p*-value 0.0233). The polynomial regression model that best fits the relationship between math anxiety and age is quadratic (Table 3). However, it is important to emphasize that the model’s R^2^ value is very small (0.0074), indicating that age explains less than 1% of the total variance in math anxiety. This suggests that while the relationship between age and math anxiety is statistically reliable, the magnitude of the effect is minimal, and the vast majority of variation in math anxiety is attributable to other unmeasured factors. These factors might include teaching methods, prior mathematical experiences, parental attitudes toward mathematics, individual differences in cognitive abilities, peer influences, and other socio-emotional variables (Dowker et al., 2016). Despite the small effect size, the significant *p*-values for all model coefficients (Table 3) support the reliability of the observed trend: math anxiety decreases with age until approximately 10 years old, then begins to increase (Figure 1). This period corresponds to the 6th grade of elementary education. This allows for the confirmation of the alternative hypothesis H2 (age significantly influences math anxiety). From Fisher’s test of correlation comparison, it follows that there are no significant differences between males and females regarding the respective correlations between math anxiety and age (i.e., the correlation between math anxiety and age is similar in males and females, with a *p*-value of 0.8051). Therefore, the null hypothesis H3 is confirmed (the correlation between age and math anxiety is not influenced by the sex).

The bilateral Pearson correlation test confirms that there is a statistically significant positive correlation between the participants’ general anxiety and their math anxiety (Pearson’s coefficient 0.1091; *p*-value < 0.0001). The linear regression model relating both variables shows a low R^2^ value (0.0119), indicating that general anxiety explains only approximately 1% of the variance in math anxiety. This small proportion suggests that while general anxiety contributes to math anxiety, the majority of variation in math anxiety stems from other sources. Potential unmeasured factors contributing to math anxiety variance include math-specific experiences (such as previous failures or successes in mathematics), teaching quality, parental attitudes specifically toward mathematics (rather than general anxiety), math self-efficacy beliefs, and domain-specific cognitive factors such as numerical abilities (Dowker et al., 2016; Ashcraft & Kirk, 2001). The small effect size is consistent with previous research showing that math anxiety, while related to general anxiety, is a distinct construct with unique predictors (Hembree, 1990; Cheng et al., 2022). However, given that the *p*-values of the slope and the independent term are less than 0.05, the model confirms the significantly increasing trend of math anxiety with general anxiety (Table 4). This confirms the alternative hypothesis of H4 (general anxiety significantly influences math anxiety). Fisher’s test for comparison of correlations applied to the male and female populations reveals that there is no significant difference between the general-anxiety–math-anxiety correlation in males and females (*p*-value: 0.1069). Thus, the null hypothesis H5 is confirmed (the correlation between general anxiety and math anxiety is not influenced by the sex).

## 5. Discussion

The study presented here makes several specific contributions to the research on math anxiety in elementary school students. First, it provides empirical data from a Spanish cultural context, addressing the geographical gap in math anxiety research, which has predominantly focused on English-speaking populations (Ashcraft & Ridley, 2005). Second, it examines not only main effects of sex, age, and general anxiety on math anxiety, but also investigates interaction effects—specifically testing whether the relationships between age/general anxiety and math anxiety differ by sex. This addresses a methodological gap identified by Dowker et al. (2016) regarding the need to understand how influential factors interact rather than operate independently. Third, by using validated instruments (STAIC and AMAS) in a Spanish sample, it contributes to the cross-cultural validation of these measurement tools. Fourth, the finding of a non-linear (quadratic) relationship between age and math anxiety extends previous research that typically treats age as a linear predictor, revealing a more nuanced developmental pattern with implications for targeting interventions at specific age groups. The context of the research is Spanish students, which differs from most of the works published in this line, which are developed in English-speaking contexts (Ashcraft & Ridley, 2005). In addition, the distribution of the participating population by sex, in total and within each grade, is homogeneous, which helps to reduce bias in the results obtained.

The results do not allow us to assume the existence of significant differences by sex in the levels of math anxiety of elementary school students. Therefore, the results are in line with studies on math anxiety in elementary education students in English-speaking countries such as the USA (Harari et al., 2013) or with an Anglo-Saxon cultural influence, such as Jamaica (Mitchell & George, 2022), or Australia (Punaro & Reeve, 2012). Thus, the results suggest that the sex stereotypes that cause the different incidence of math anxiety in females and males (Rossi et al., 2022; Justicia-Galiano et al., 2023; Vos et al., 2023) have not yet appeared, at least in the geographical context of this study, among the under-12 student population. Likewise, the results differ from those presented in the literature in adolescent and adult populations. Indeed, it is common for the literature to find significant differences in math anxiety levels between females and males in adult populations (Kvedere, 2014; Primi et al., 2014; Ferguson et al., 2015; Rossi et al., 2022; Justicia-Galiano et al., 2023). This observation would support the idea that, before the age of 12, the consequences of cultural sex stereotypes on the incidence of math anxiety in children have not yet appeared. However, it would be necessary to contrast this hypothesis with contextualized studies in populations of Spanish adolescents and students.

There are divergences between the results obtained here and those of other works that find differences between the math anxiety of females and males. These divergences can be explained by the notable cultural differences in the respective contexts, such as the Asian context (Yu et al., 2024). In contrast, Arnal-Palacián et al. (2022) report differences between female and male elementary school students in their levels of math anxiety. The divergence with the present study could be explained by the different measurement instrument used and by the influence of confinement due to the COVID-19 pandemic. But it also suggests that there may be other sociodemographic factors within Spain that influence the levels of math anxiety, and that should be identified.

Moreover, while our finding of a quadratic age trend is statistically reliable, the minimal variance explained (R^2^ = 0.0074) has important implications for interpreting developmental changes in math anxiety. The small effect size indicates that chronological age itself—and the curricular and developmental changes associated with it—exerts only a weak influence on math anxiety when considered in isolation from other factors. This suggests that the observed increase in math anxiety around age 10 may not be primarily driven by universal developmental processes or curriculum complexity per se, but rather by how individual students interact with these factors based on their unique constellation of experiences, abilities, self-beliefs, and social contexts. Two students of the same age exposed to the same curriculum may exhibit vastly different math anxiety levels depending on these individual differences. Consequently, age-based interventions (e.g., modifying curriculum difficulty at specific grade levels) are unlikely to be sufficient without also addressing individual variability in the factors that make students more or less vulnerable to math anxiety at each developmental stage. Educators should interpret our age findings as identifying when students may be at heightened risk for math anxiety increases, but must recognize that effective intervention requires identifying and addressing the student-specific factors that determine who actually experiences these increases.

In this work, age has also been identified as an influential factor in the levels of math anxiety (Table 3), with no differences between females and males in this regard. This is consistent with the previous observation that sex differences in math anxiety do not occur in the same way in children under 12 years of age and in adolescents and adults. In fact, it is shown here that the evolution of math anxiety levels with age is not linear, but quadratic. This implies that math anxiety decreases with increasing age until about 10 years old, when it begins to grow (Figure 1). Several interpretative hypotheses may account for this pattern. One possibility is that the increasing complexity of mathematical content around age 10—including the introduction of algebraic language, rational numbers, and more abstract mathematical concepts—creates new sources of anxiety for students who may have successfully managed simpler arithmetic tasks (Ashcraft & Kirk, 2001). Alternatively, changes in teaching methodology or assessment practices in upper elementary grades may contribute to increased anxiety (Wang et al., 2015; Dondio et al., 2023). A third hypothesis is that this age coincides with developmental changes in self-awareness and social comparison processes, leading students to become more conscious of their mathematical abilities relative to peers (Wigfield & Meece, 1988). However, these interpretations are speculative and require direct investigation. Future research should explicitly examine curricular content, teaching practices, and cognitive-developmental factors at different elementary school grades to identify the mechanisms driving the observed quadratic trajectory of math anxiety.

An important consideration when interpreting our findings is the distinction between statistical significance and practical significance. While we found statistically significant relationships between math anxiety and both age (R^2^ = 0.0074) and general anxiety (R^2^ = 0.0119), the extremely small proportions of variance explained suggest that these relationships have limited practical significance for educational intervention. Explaining less than 1% of variance means that even if educators could perfectly manipulate age-related factors or reduce general anxiety to zero, math anxiety levels would decrease only minimally. This has direct implications for intervention design: programs focused solely on age-appropriate curricular adjustments or general anxiety reduction are unlikely to substantially reduce math anxiety without addressing other, more influential factors. The minimal variance explained also suggests that our study has identified reliable trends (the quadratic age pattern, the positive general anxiety relationship) that are theoretically meaningful for understanding math anxiety development, but that practitioners and policymakers should not overestimate the impact of these specific factors in isolation. The substantial unexplained variance (>99%) indicates that math anxiety is determined by a complex constellation of factors beyond those measured here, and effective interventions will likely need to be multifaceted, addressing domain-specific factors such as math self-efficacy, prior mathematical experiences, instructional quality, and math-specific parental attitudes.

Furthermore, this result on the influence of age on math anxiety extends the results obtained by Arnal-Palacián et al. (2022), who do not analyze age as an explanatory variable. On the other hand, this result does not agree with other works, such as that by Yu et al. (2024) which does not find a significant evolution in the levels of math anxiety with age. Again, the cultural difference of the populations could explain this divergence of results (Brown et al., 2020).

It has also been shown that there is a positive and moderate correlation between general trait anxiety and math anxiety. In this case, the correlation is linear (Table 4). This indicates that a higher level of general anxiety leads to a higher level of math anxiety, but that math anxiety is not completely explained by trait general anxiety. This is in line with preceding research (Punaro & Reeve, 2012; Hill et al., 2016; Cheng et al., 2022). It is also found that the correlation between general anxiety and math anxiety does not depend on the sex of the participants. This agrees with the results of Devine et al. (2012), obtained in a British context. It also extends the results of Hill et al. (2016), who did not analyze sex differences in the correlation between general anxiety and math anxiety. All this confirms that the introduction of measures to control general anxiety in schoolchildren helps control math anxiety (Hill et al., 2016).

## 6. Conclusions

The participating Spanish elementary school students report moderate mean levels of math anxiety. Furthermore, there are no significant differences between males and females in the mean levels of math anxiety. However, age is an explanatory variable for math anxiety levels, such that it decreases with age until around the age of 10, when math anxiety increases with age. The way in which the age of the participants influences their math anxiety is the same for females and males. Also, the level of trait general anxiety of the participants correlates positively with the level of math anxiety and does so without significant differences between males and females. However, it should be noted that while these relationships are statistically significant, they explain minimal variance in math anxiety, indicating limited practical significance for intervention design when these factors are considered in isolation.

The results obtained suggest that specific measures for the control of math anxiety in Spanish schools are warranted, but the minimal variance explained by the factors we examined indicates that effective interventions must extend beyond general anxiety management and age-appropriate curricular adjustments. Future intervention programs should incorporate domain-specific components targeting math self-efficacy, addressing math-specific parental and teacher attitudes, and modifying instructional practices known to influence math anxiety. Given the positive correlation between general anxiety and math anxiety, these measures could consist, at least in part, of general anxiety control measures. These could be carried out for students in general in academic tutoring sessions. No significant differences by sex were found, from which it follows that any differences that may appear in this respect at later stages may be due to cultural aspects or the presence of stereotypes that, in any case, should be identified and corrected. However, it does not seem necessary to take this type of measure in elementary education, at least in the Spanish cultural context. On the other hand, it is worrying that there is an increasing trend in the levels of math anxiety at the end of the elementary education stage. This pattern raises questions about the methodological and evaluation strategies being used in mathematics education in the 10–12 age group and warrants further investigation to determine whether curricular or pedagogical adjustments might be beneficial. It is surely necessary to reinforce the manipulative and meaningful character of these strategies, as well as the mechanisms to increase motivation. This favors a more lasting and deeper learning of mathematics, and students will have greater self-confidence in handling eventually complex concepts.

Finally, the study has several important limitations that must be acknowledged. First and most significantly, the use of a convenience sample from a single Spanish school substantially limits the generalizability of our findings. Spain exhibits considerable sociodemographic variability across regions, including differences in socioeconomic status, educational resources, teaching methodologies, cultural attitudes toward mathematics, and regional policies on education. Students in the Castile and León region may experience different educational contexts compared to students in other autonomous communities such as Andalusia, Catalonia, or the Basque Country. Additionally, the single-school design means our findings may reflect school-specific factors—such as particular teaching approaches, institutional culture, or student composition—that do not represent even the broader Valladolid educational context, let alone Spain as a whole. This limitation is particularly important given that our study found no sex differences in math anxiety, a finding that contrasts with some other Spanish research. The discrepancy could reflect genuine regional or school-level differences in how gender stereotypes influence math anxiety, underscoring the need for multi-site studies. Future research should employ stratified random sampling across multiple schools and regions within Spain to determine whether the patterns observed here generalize or vary systematically by geographic, socioeconomic, or institutional characteristics. Such research would also allow investigation of contextual moderators that may explain conflicting findings in the Spanish literature. This would allow for a comparison of the results obtained here and, if applicable, identify the existence of differences in mathematical anxiety behavior among students from different geographical areas. With the same objective, it is suggested to carry out a comparative study on the incidence of mathematical anxiety in different regions.

A second major limitation concerns the restricted set of predictor variables included in our models. While we examined sex, age, and general anxiety, the minimal variance explained (R^2^ < 0.012 for all models) clearly indicates that the most influential determinants of math anxiety were not measured in this study. Based on the existing literature, we can identify several categories of variables that likely account for substantial portions of the unexplained variance. First, cognitive and affective variables such as math self-efficacy (students’ confidence in their mathematical abilities), prior math achievement, domain-specific anxiety about tests versus learning situations, and attributional styles (whether students attribute math failures to stable vs. unstable causes) have been shown to be strong predictors of math anxiety. Second, social and environmental variables including parental attitudes specifically toward mathematics (not general anxiety), parents’ own math anxiety levels which can be transmitted to children, teacher math anxiety and teaching practices, peer influences, and classroom climate around mathematics are documented influences on student math anxiety. Third, instructional variables such as pedagogical approach (traditional versus reform-based instruction), frequency of active learning versus rote practice, formative versus summative assessment emphasis, and availability of individualized support have been associated with math anxiety levels.

Future research should employ more comprehensive models that include these domain-specific predictors alongside the demographic and general psychological variables examined here. Multi-level modeling approaches would be particularly valuable, allowing researchers to distinguish individual-level factors (self-efficacy, prior achievement) from classroom-level factors (teaching practices) and school-level factors (resources, institutional culture). Such research would provide more actionable insights for intervention development by identifying the factors with the strongest practical impact on math anxiety.

Additionally, it would be advisable to expand the study conducted here to student populations with a wider age range. This would allow for a more in-depth study of the influence of age on mathematical anxiety. It is also suggested to complement the obtained results with the conclusions of a qualitative study on math anxiety among elementary school students. This qualitative analysis would help identify the reasons perceived by the students that explain the math anxiety exhibited.

## Figures and Tables

**Table 1:** Distribution of participants of each sex within each grade.

Grade	Number of Participants	Males (%)	Females (%)
3rd	46	52.2	47.8
4th	45	48.9	51.1
5th	48	43.8	56.2
6th	46	47.8	52.2

**Table 2:** Descriptive statistics of the responses.

	Mean	SD	Coefficient of Variation (%)
General anxiety (out of 3)	1.79	0.79	43.86
Math anxiety (out of 5)	2.02	1.28	63.68

**Table 3:** Quadratic regression model to explain math anxiety from the age of participants.

	Estimated	Standard Error	*t*-Statistic	*p*-Value
Independent term	7.4946	1.8746	4.00	<0.0001
Linear coefficient	−1.0974	0.3932	−2.79	0.0053
Quadratic coefficient	0.0540	0.0204	2.65	0.0081

**Table 4:** Linear regression model to explain math anxiety from general anxiety.

	Estimated	Standard Error	*t*-Statistic	*p*-Value
Independent term	1.6975	0.0777	21.85	<0.0001
Slope	0.1696	0.0379	4.47	<0.0001

## Data Availability

Data are available upon reasonable request to the corresponding author.

## Appendix A

STAIC T-Anxiety questionnaire (1 to 3 Likert scale, where 1 means almost never, 2 means sometimes, and 3 means frequently).

1. I worry about making mistakes.
2. I often feel like crying.
3. I feel unhappy.
4. I have trouble making up my mind.
5. It is difficult for me to face my problems.
6. I worry too much.
7. I get upset at home.
8. I am shy.
9. I feel troubled.
10. Unimportant thoughts run through my mind and bother me.
11. I worry about school.
12. I have trouble deciding what to do.
13. I notice my heart beats fast.
14. I am secretly afraid.
15. I worry about my parents.
16. My hands get sweaty.
17. I worry about things that may happen.
18. It is hard for me to fall asleep at night.
19. I get an unpleasant feeling in my stomach.
20. I worry about what others think of me.

AMAS questionnaire (1 to 5 Likert scale, where 1 is very low anxiety; 2 is low anxiety, 3 is intermediate anxiety, 4 is high anxiety, 5 is very high anxiety).

How anxious would you feel…

21. … having to use the tables in the back of a math book?
22. … thinking about the math exam the day before doing it?
23. … watching a teacher doing a difficult operation on the blackboard?
24. … doing a math exam?
25. … if you have many difficult problems as homework for the next lecture?
26. … listening to a lesson in math class?
27. … listening to another student explain a math problem?
28. … if someone asks you questions in math class?
29. … starting a new math book chapter?
